# Low-Density Lipoprotein Cholesterol Is a Possible Blood Biomarker of Schizoid Personality Traits among Females

**DOI:** 10.3390/jpm12020131

**Published:** 2022-01-19

**Authors:** Kohei Hayakawa, Motoki Watabe, Hideki Horikawa, Mina Sato-Kasai, Norihiro Shimokawa, Tomohiro Nakao, Takahiro A. Kato

**Affiliations:** 1 Department of Neuropsychiatry, Graduate School of Medical Sciences, Kyushu University, Maidashi 3-1-1, Higashi-ku, Fukuoka 812-8582, Japan; ko.hayakaw@gmail.com (K.H.); molamola@sol.dti.ne.jp (H.H.); ks_mina@hotmail.com (M.S.-K.); nolisup@yahoo.co.jp (N.S.); nakao.tomohiro.275@m.kyushu-u.ac.jp (T.N.); 2 School of Business, Monash University Malaysia, Jalan Lagoon Selatan, Bandar Sunway, Subang Jaya 47500, Malaysia; motoki.watabe@monash.edu

**Keywords:** schizoid personality disorder, schizophrenia, low-density lipoprotein cholesterol (LDL-C), loneliness, solitude, social isolation, hikikomori, trust game, social decision making

## Abstract

Lower serum levels of low-density lipoprotein cholesterol (LDL-C) have been suggested to indicate higher suicide risk and various psychiatric symptoms. Previously, we reported that lower serum LDL-C levels are associated with loneliness, social phobia, isolated life with little social support, and lower trust in others among young non-clinical females. Thus, we hypothesize that schizoid personality traits may be associated with lower serum LDL-C. We here verified this hypothesis using non-clinical data and clinical data with schizophrenia. Using the database from the Midlife in Japan (MIDJA), a cohort of residents living in Tokyo, we analyzed whether schizoid-related interpersonal characteristics were associated with LDL-C. In addition, we assessed the association between blood biomarkers including LDL-C and schizoid personality traits in 101 adult non-clinical volunteers. Finally, we evaluated the interaction between LDL-C and social decision making of patients with schizophrenia. In female non-clinical volunteers, serum LDL-C level was a predictive factor and negatively correlated with schizoid personality traits. Female patients with schizophrenia, whose serum LDL-C levels were lower, tended not to trust other females. The present findings suggest that LDL-C may influence schizoid personality traits in females, which provide a basis for further investigation into the biological aspects of schizoid personality disorder.

## 1. Introduction

Schizoid personality disorder is known as one of the personality disorders that are often seen before the onset of schizophrenia [1]. Schizoid personality disorder is defined as “a pervasive pattern of detachment from social relationships and a restricted range of expression of emotions in interpersonal settings” in the Diagnostic and Statistical Manual of Mental Disorders, Fourth Edition (DSM-IV) [2], with a prevalence of approximately 1% [3,4] and a gender difference in the prevalence with more males than females [5]. “A pervasive pattern of detachment from social relationships”, which is characteristic of schizoid personality disorder, often leads the patient to become lonely and isolated. Loneliness and social isolation have become one of the major social issues in social life. Various factors such as changes in family structure including an increase in the lifelong unmarried rate and the shift to nuclear families, diversification of values and relationships in response to social changes, and life transitions such as divorce and bereavement have contributed to people facing loneliness. “Hikikomori” (a severe form of social withdrawal) or “Kodoku-shi” (death by loneliness) has become one of the most serious problems of modern society in Japan [6], and these problems has been spreading worldwide [7,8,9,10]. Lonely young people tend to use the Internet as an emotional support tool [11], which is closely related to problems such as Internet addiction. In the United Kingdom, about 14% of the population reported feeling lonely often or all the time, which led to the creation of the new position of Minister for Loneliness in January 2018, as loneliness has a more negative impact on health than smoking [12]. Although loneliness is often highlighted as a social problem, it is actually related to a variety of physical and mental problems. In relation to physical problems, high loneliness is associated with an increased incidence of coronary heart disease [13], and social isolation is regarded to result in negative health conditions and is associated with increased mortality [14,15]. In relation to mental problems, loneliness is correlated with cognitive dysfunction in the elderly [16] and the degree of depression [17]. Loneliness may be interconnected with mental functioning and behavioral characteristics, and it has also been linked to suicide [18]. Moreover, in the midst of the Coronavirus Disease 2019 (COVID-19) pandemic worldwide, many people are experiencing loneliness, and there is concern that this may lead to an increase in patients with depression, hikikomori, and suicides [19,20]. In the background of the association between loneliness and various physical and mental problems, it has been speculated that there is a biological basis for the association between loneliness and dysregulation of adrenal cortical hormone secretion [21], and inflammatory cytokine responsiveness to mental stress [22]. However, many of the details remain unknown about the biological basis.

More than a quarter of a century ago, Lindberg et al. reported that there was an association between increased suicide risk and lower serum levels of low-density lipoprotein cholesterol (LDL-C) [23], and that lowering LDL-C levels may increase deaths other than from disease (i.e., deaths from accidents, suicide, or violence) [24]. Subsequently, it has become increasingly clear that low LDL-C is also associated with psychiatric symptoms such as anxiety and impulsivity [25], depressive symptoms [26], depressive states, low cognitive performance [27], and neuroticism [28]. A recent meta-analysis has shown a U-shaped relationship between depression and serum LDL-C levels [29]. These reports suggest that abnormal levels of LDL-C may not only be a risk factor for the development of physical diseases but may also be involved in some way in the development of various psychiatric and behavioral symptoms and mental disorders. Previously, we reported that lower serum LDL-C levels are associated with higher levels of loneliness and social phobia, living an isolated life with less social support, and less trust in others in young non-clinical volunteer females [30]. This hypothesizes that serum LDL-C levels may be related to specific personality tendencies and interpersonal characteristics, especially schizoid personality.

In the present study, we verified this hypothesis using non-clinical data from volunteers and clinical data from patients with schizophrenia. We first examined the relationship of serum LDL-C levels to schizoid personality by gender using database from the Midlife in Japan (MIDJA), which is a cohort of residents living in Tokyo. The results of the examination showed that the interpersonal characteristics associated with low serum LDL-C levels in females were most likely to reflect their schizoid personality traits. Each personality disorder has a biological pathophysiological basis [31], and schizoid personality disorder is no exception. Therefore, based on the findings of the MIDJA data analysis, we next investigated the relationship between serum LDL-C levels and schizoid personality traits in student-age non-clinical volunteers by gender. Finally, a personal computer (PC)-based trust game was conducted with patients with schizophrenia to investigate the relationship between serum LDL-C levels and social decision making of patients with schizophrenia.

## 2. Materials and Methods

### 2.1. Study 1: The MIDJA Data Analysis

In the MIDJA survey, 1027 adults were randomly recruited from residents of 23 wards in Tokyo (*N* = 1027, 505 males and 522 females, aged 30 to 79). The questionnaire was administered to the participants from April to September 2008. Data were collected regarding socio-demographic characteristics (age, gender, marital status, and educational status) and a self-rated questionnaire (independence/interdependence, personality traits, sense of control, goal orientations, social support, family obligation, and social responsibility) completed by participants. From January 2009 to April 2010, biomarker data including blood assays were obtained from a portion of participants (382 out of 1027, 168 males and 214 females) at a clinic near the University of Tokyo (Biomarker Project). The results of these surveys are publicly available by the Inter-university Consortium for Political and Social Research (ICPSR), and the following analysis was conducted using the published MIDJA survey data.

Using the MIDJA data, we assessed the correlations between serum LDL-C levels and six personality traits (‘neuroticism’; ‘extraversion’; ‘openness to experience’; ‘conscientiousness (4 items)’; ‘agreeableness’; and ‘agency’) and various problems of close interpersonal relationships with friends, spouse/partner, and family as follows: (1) problems with friends (‘friendship support scale’; ‘support from/given to friends’; ‘strain from/given to friends’; and ‘affectual solidarity from/given to friends’), (2) problems with spouse/partner (‘marital risk’; ‘spouse/partner disagreement’; ‘spouse/partner decision making’; ‘support from/given to spouse/partner’; ‘strain from/given to spouse/partner’; and ‘affectual solidarity from/given to spouse/partner’), and (3) problems with family (‘support from/given to family’; ‘strain from/given to family’; and ‘affectual solidarity from/given to family’) for each gender.

### 2.2. Study 2: Study on Student-Age Non-Clinical Volunteers

#### 2.2.1. Subjects

We selected the university campus as an appropriate place to recruit participants and advertise our study. Participants mostly consisted of university students and were recruited by print advertisement, “CALL FOR MENTALLY AND PHYSICALLY HEALTHY VOLUNTEERS”, on the campus of Kyushu University from February to May 2014. Socio-demographic information of all participants was collected; then, a medical doctor assessed whether the participants were healthy or not. The inclusion criteria of the present study were as follows: (1) proficient in Japanese and (2) more than 17 years old. The exclusion criteria were participants with severe heart, liver, or kidney diseases.

#### 2.2.2. Procedure

After obtaining written informed consent, we administered self-rating questionnaires including the Structured Clinical Interview for DSM-IV Personality Disorders Personality Questionnaire (SCID-II/PQ), and the following blood tests using non-fasting venous blood samples: serum LDL-C and high-density lipoprotein cholesterol (HDL-C), serum total bilirubin and uric acid (UA), and serum high-sensitivity C-reactive protein (hsCRP) and plasma fibrin degradation products (FDP). We adopted these biomarkers as measured items because of their easy measurement in normal clinical settings and comparison with the MIDJA survey, in which some of the biomarkers were also adopted.

#### 2.2.3. Self-Rating Questionnaires

The serious interpersonal problems that characterize schizoid personality disorder are mainly related to isolation and loneliness, which are naturally related to the sense of trust and fear of others, social phobia, and the social networks actually built with others. In addition, in today’s Information Technology society, isolated living and the degree of dependence on the Internet are considered to have an important relationship. Therefore, in this study, we measured the following psychological rating scales, which are probably related to the mentality of patients with schizoid personality disorder and their real lives. SCID-II/PQ includes a 119-item questionnaire version of the SCID-II, on which items are answered using a “yes” (1) or “no” (0) response format [32]. Items corresponding to each personality disorder are summed for a total score defined as an index of each personality trait in this study. Schizoid personality traits were evaluated with SCID-II/PQ (Max. 7 points). Furthermore, we administered the following questionnaires: the Preference for Solitude Scale (PSS), assessing one’s preference for solitude [33]; the Revised University of California, Los Angeles Loneliness Scale (R-UCLA), assessing one’s degree of loneliness [34,35]; the Yamagishi and Yamagishi’s trust scale (YYS), measuring respondents’ estimation of others’ trustworthiness [36]; the Internet Addiction Test (IAT), assessing the degree to which Internet use affects daily routine, social life, productivity, sleeping pattern, and feelings [37]; the Lubben Social Network Scale-6 (LSNS-6), gauging social isolation by assessing the number of people in one’s social network with whom one has social contact and social support [38,39]; the Multidimensional Scale of Perceived Social Support (MSPSS), measuring the perceived adequacy of support from family, friends, and significant others [40]; and the Mini Social Phobia Inventory (MINI-SPIN), having good efficiency in distinguishing individuals with generalized social anxiety in a health care setting [41,42].

### 2.3. Study 3: Study on Patients with Schizophrenia

#### 2.3.1. Subjects

We recruited patients diagnosed and treated for schizophrenia by an experienced psychiatrist in a community psychiatric hospital as participants in the study. The inclusion criteria of the present study were as follows: (1) proficient in Japanese and (2) more than 17 years old. The exclusion criteria were participants with severe heart, liver, or kidney diseases.

#### 2.3.2. Procedure

After obtaining written informed consent, all participants played a PC-based trust game and provided a non-fasting venous blood sample. We measured the same blood biomarkers as on the second study on student-age non-clinical volunteers.

#### 2.3.3. PC-Based Trust Game

Based on our previous research, we assessed participants’ trusting behavior toward others by a PC-based trust game [43]. In this two-player game, each player is first given an explanation of the rules of the game and receives 1300 yen (about 12 US dollars). After that, the first player decides how much of the 1300 yen to give to the second player (partner) and evaluates the partner’s attractiveness and trustworthiness with a score by looking at the partner’s picture displayed on the computer screen (both scores are from 0 to 9). The amount of money (i.e., monetary scores) decided upon is tripled and given to the partner. Then, the second player decides whether to divide the money equally with the first player or take away the entire amount. In this PC experiment, all participants are actually the first player, and the second player is a virtual player only on the computer screen. The participants are not given any information about their partners, except for a photo of their face, including head and shoulders with a neutral expression. The photo of the second player is chosen from professional fashion models or ordinary people. In the present study, 40 photos were employed (10 each of professional male fashion models, professional female fashion models, lay males, and lay females). The first player was not informed of their partner’s decision. The decision of how much money the first player would give to the second player reflected the level of trust in the second player. After the experiment, each participant was paid an amount of money in real money as a reward, corresponding to the outcome of a randomly selected game from all the games.

### 2.4. Statistical Analyses

We performed statistical analyses by using SPSS, version 22 (SPSS, Chicago, IL, USA). All analyses were stratified by participant gender. First, we analyzed the data from the MIDJA. Correlation analyses were performed between serum LDL-C levels and six personality traits and various problems of close interpersonal relationships with friends, spouse/partner, and family to calculate Spearman’s correlation coefficient (ρ). Second, correlation analyses were performed between schizoid personality scores of SCID-II/PQ, self-administered questionnaires, and blood biomarkers to calculate Spearman’s correlation coefficient (ρ) on student-age non-clinical samples. Third, regression analyses were performed with the stepwise method for efficient biomarker detection and determination of whether serum LDL-C levels predicted schizoid personality scores. We included six independent variables: LDL-C, HDL-C, total bilirubin, UA, FDP, and hsCRP. The dependent variable was schizoid personality score. To avoid the issue of multicollinearity, we chose the stepwise option and confirmed that the scores of condition indices were below fifteen for each final model. Finally, correlation analyses were performed between serum LDL-C levels and three types of social decision making (monetary scores, ratings of trustworthiness, and ratings of attractiveness) in a trust game to calculate Pearson’s correlation coefficient (r) or Spearman’s correlation coefficient (ρ) on patients with schizophrenia. We used a two-tailed significance level of *p* = 0.05.

## 3. Results

### 3.1. The MIDJA Data Analysis (Study 1)

The relationships between serum LDL-C levels, six personality traits, and “problems of close interpersonal relationships” were shown in Table 1. In males, the results of correlation analysis showed that serum LDL-C levels were positively correlated with ‘openness to experience’, and negatively correlated with ‘friendship support scale’ (ρ = 0.190, *p* = 0.014; and ρ = −0.159, *p* = 0.039, respectively). In females, the results of correlation analysis showed that serum LDL-C levels were negatively correlated with ‘spouse/partner disagreement’ (ρ = −0.245, *p* = 0.002), and positively correlated with ‘affectual solidarity given to spouse/partner’ and ‘support given to family’ (ρ = 0.181, *p* = 0.024; and ρ = 0.169, *p* = 0.034, respectively). These results suggest that low serum LDL-C levels are related to poor and problematic interpersonal relationships in females. Schizoid personality disorder is known as a personality disorder characterized by scarcity and problems in interpersonal relationships, and the above aspects of MIDJA are often observed in persons with schizoid personality. 

### 3.2. Study on Student-Age Non-Clinical Volunteers (Study 2)

Thus, we next examined the relationship between serum LDL-C levels and schizoid personality traits in student-age non-clinical volunteers. Participants’ demographic details are shown in Table 2. We analyzed each group of student-age non-clinical volunteers separately by gender. The relationships between schizoid personality scores, psychometrics, and blood biomarker levels are shown in Table 3. In males, the results of correlation analysis showed that schizoid personality scores were positively correlated with R-UCLA and negatively correlated with serum total bilirubin levels (ρ = 0.374, *p* = 0.010; and ρ = −0.311, *p* = 0.035, respectively). We conducted multiple regression analysis using the stepwise method, with schizoid personality score as the dependent variable and blood biomarkers as independent variables for efficient biomarkers detection; however, no statistically significant independent variables were detected (Table 4). In females, the results of correlation analysis showed that schizoid personality scores were positively correlated with the Preference for Solitude Scale (PSS) and R-UCLA, and they were negatively correlated with serum LDL-C levels (ρ = 0.498, *p* < 0.001; and ρ = 0.271; *p* = 0.046; and ρ = −0.307, *p* = 0.022, respectively). We conducted multiple regression analysis using the stepwise method in the same way as for males. Regression analyses with the stepwise method revealed that schizoid personality score was predicted by only serum LDL-C levels (β = −0.312, *p* = 0.02, condition index = 8.825) (Table 4).

### 3.3. Study on Patients with Schizophrenia (Study 3)

The trust game is one of the economic games that can evaluate tendencies of human social behaviors directly and quantitively. Thirty-three patients with schizophrenia including 18 males (mean age = 40.6, 95% confidence interval (CI)= 36.3–44.8) and 14 females (mean age = 41.0, 95% CI = 37.5–44.5) participated in the PC-based trust game. In male participants with schizophrenia, the results of correlation analysis did not show any statistically significant correlation between serum LDL-C levels and each decision in a trust game. In female participants with schizophrenia, the results of correlation analysis showed that serum LDL-C levels were positively correlated with the trustworthiness of female partners (r = 0.546, *p* = 0.043) (Table 5).

## 4. Discussion

In the present study, we first analyzed the MIDJA data by gender, and we found that low serum LDL-C levels are associated with poor and problematic interpersonal relationships. Based on this analysis, we hypothesized that poor and problematic interpersonal relationships reflect the characteristics of schizoid personality disorder. Next, we conducted an analysis of the data for student-age non-clinical volunteers and found that female participants with lower serum LDL-C levels tended to have higher schizoid personality scores. On the other hand, no such association between serum LDL-C levels and schizoid personality scores was found in male participants. Finally, we conducted a PC-based trust game with patients with schizophrenia and examined the correlation with serum LDL-C levels, and we found that female patients with lower serum LDL-C levels tended not to trust their female partners.

To our knowledge, this is the first study showing the association between serum LDL-C levels and schizoid personality traits in young non-clinical females and between serum LDL-C levels and the decision-making tendencies of female patients with schizophrenia. Boundaries between normal and abnormal personality are artificial and a matter of controversy [44]; thus, personality traits and disorders form a continuum, and it is difficult to clearly discriminate between personality “disorders” and personality “traits” within a normal limit. Healthy people and patients with psychiatric disorders including schizophrenia bear substantive similarities: for example, overlap in mental states, behaviors, and personalities. In addition, healthy people can have various personality traits corresponding to each particular personality disorder, although their personalities do not reach the level of “personality disorders”. Therefore, it would be meaningful to investigate characteristics of particular personality traits using both clinical patients and non-clinical persons to deepen our understanding of the corresponding personality disorders.

In spite of evidence that individuals with schizoid personality disorder are characterized by serious social, psychiatric, and socioeconomic dysfunction [45], the biological pathophysiology of schizoid personality disorder remains to be fully elucidated. Schizoid personality disorder, along with avoidant personality disorder and schizotypal personality disorder, is a common premorbid personality of schizophrenia [1]. The symptoms include those common to the negative symptoms of schizophrenia, such as the following: little desire for relationships, choosing solitary activities, never has strong feelings, no close friends, little desire for sexual relationships, indifferent to praise or criticism, and odd speech [46]. The characteristics of social anhedonia that underlie these symptoms are not just avoidance of social relationships but a genuine disinterest in social relationships. People with high social anhedonia are considered to be at high risk for developing schizophrenia spectrum disorders [47]. Focusing on the symptom similarity between schizoid personality disorder and schizophrenia, a relevant biological pathological basis of LDL-C is assumed in the common areas of these two disorders, i.e., problems reflecting social anhedonia such as scarcity and problems with interpersonal relationships. Biological investigations of premorbid personality traits could help us deepen our understanding of schizoid and schizophrenia pathophysiology, assess risk of developing schizophrenia, and prevent schizophrenia.

The relationship between schizoid personality traits and blood biomarkers shown in the present study has a gender difference. A previous study has shown that schizoid personality disorder is a predictor of whether psychotherapy is effective in only female patients [48], suggesting that gender differences in schizoid tendency may affect therapeutic strategies. These gender differences may reflect the nature of the pathological difference between schizoid males and schizoid females. These differences may be caused by multiple factors such as lifestyle differences, cultures that accept gender differences in thought, cognition, behaviors, intrapersonal masculinity and femininity, and sex hormones and estrous cycles, which can influence mental function, including mood and emotion [49].

Several limitations exist in the present study. First, the subjects who participated in this study as student-age non-clinical volunteers were recruited from only a certain region and an institution, and their number is small. The number of patients with schizophrenia participated in the study was also small. The possibility of selection bias cannot be ruled out. However, no previous study has reported that serum LDL-C levels are associated with schizoid personality traits and social decision making in a PC-based trust game, and we believe that this study has strong importance. Second, because structured diagnostic interviews were not conducted with the non-clinical volunteers, we cannot rule out the possibility that some of the participants in this study may have suffered from some kind of psychiatric disorder. Similarly, the participants in study 2 were not diagnosed with schizoid personality disorder by structured diagnostic interviews. Instead, they were assessed for schizoid personality traits by taking the SCID-II/PQ. In future studies, it will be necessary to assess the comorbidity of psychiatric disorders by conducting structured interviews such as the Structured Clinical Interview for the DSM. Third, the present cross-sectional study could not evaluate the causal relationship between schizoid personality traits and serum LDL-C levels. In addition, this pilot study suggests that LDL-C may be a biological marker of schizoid personality traits among females, and even though schizoid personality traits are one of the premorbid personality traits of schizophrenia, it does not necessarily directly imply that females with low serum LDL-C are at higher risk of developing schizophrenia. To clarify this point, it is necessary to consider that the further long-term longitudinal observations of females with low serum LDL-C will be needed. Fourth, there may also be confounding factors such as comorbid physical diseases such as thyroid dysfunction, tobacco, alcohol, and dietary and exercise habits. Hence, further research is needed to validate the present results and to clarify the role of LDL-C in various interpersonal relationships and schizoid personality.

## 5. Conclusions

In conclusion, based on the significant findings in the present study, we propose the hypothesis that low LDL-C is a possible blood biomarker of schizoid personality traits among young female adults. To elucidate the role of LDL-C in the development of schizoid personality traits and schizophrenia, further studies are required.

## Figures and Tables

**Table 1:** The relationships between serum LDL-C levels, six personality traits, and “problems of close interpersonal relationships”.

	Serum LDL-C Levels					
	Male			Female		
	ρ	*p*	*n*	ρ	*p*	*n*
personality traits						
neuroticism	0.000	1.00	167	−0.127	0.064	214
extraversion	0.106	0.17	167	0.026	0.71	214
**openness to experience**	** 0.190 **	** 0.014 **	**167**	0.044	0.53	214
conscientiousness (4-itmes)	0.121	0.12	167	−0.034	0.62	214
agreeableness	0.141	0.07	167	−0.003	0.97	214
agency	0.124	0.11	167	0.044	0.52	214
**friendship support scale**	** −0.159 **	** 0.039 **	**168**	0.090	0.19	213
support						
from friends	0.069	0.37	167	0.125	0.068	214
given to friends	0.022	0.78	166	0.069	0.32	213
strain						
from friends	−0.044	0.58	166	0.078	0.26	214
given to friends	−0.107	0.17	167	0.055	0.43	212
affectual solidarity						
from friends	0.108	0.17	167	0.032	0.64	214
given to friends	0.092	0.24	166	−0.009	0.90	212
marital risk	−0.012	0.89	140	−0.153	0.052	162
**spouse/partner disagreement**	−0.010	0.91	138	**−0.245**	**0.002**	**159**
spouse/partner decision making	−0.062	0.48	135	0.132	0.097	160
support						
from spouse/partner	−0.027	0.76	138	0.135	0.089	160
given to spouse/partner	−0.063	0.47	136	0.146	0.068	158
strain						
from spouse/partner	0.047	0.59	137	−0.133	0.092	161
given to spouse/partner	0.099	0.25	136	−0.117	0.14	157
**affectual solidarity**						
from spouse/partner	−0.062	0.47	138	0.150	0.057	162
**given to spouse/partner**	−0.067	0.44	135	**0.181**	**0.024**	**157**
**support**						
from family	0.118	0.21	115	−0.012	0.88	156
**given to family**	0.089	0.34	115	**0.169**	**0.034**	**157**
strain						
from family	−0.035	0.71	115	0.062	0.44	157
given to family	−0.084	0.37	115	−0.021	0.79	156
affectual solidarity						
from family	0.098	0.30	115	−0.015	0.85	158
given to family	0.113	0.23	115	0.144	0.073	156

ρ: Spearman’s correlation coefficient, significant *p* values are shown in bold type.

**Table 2:** Demographic data.

	Male	Female	*p* Value
*n*	46	55	
age, median (IQR)	21.0 (4.0)	21.0 (2.0)	0.43 ^b^
nationality			
Japanese	44	55	0.12 ^a^
not Japanese (Asians)	2	0	
cohabitation			
living alone	33	35	0.39 ^a^
not living alone	13	20	
status			
current student	44	55	0.37 ^a^
worker	2	0	

^a^ Chi-squared test, ^b^ Welch’s t test or Mann–Whitney U-test.

**Table 3:** The relationships between schizoid personality scores, psychometrics, and blood biomarker levels.

	Schizoid Personality Score			
	Male		Female	
	ρ	*p*	ρ	*p*
psychometrics				
**PSS**	0.251	0.092	**0.498**	**<0.001**
**UCLA**	**0.374**	**0.010**	**0.271**	**0.046**
YYS	−0.201	0.18	−0.128	0.35
IAT	0.216	0.15	0.123	0.37
**LSNS-6**	−0.208	0.17	**−0.316**	**0.019**
MSPSS	−0.183	0.22	−0.192	0.16
MINI-SPIN	0.204	0.17	0.103	0.46
blood biomarkers				
HDL-C	−0.027	0.86	0.041	0.77
**LDL-C**	−0.051	0.74	**−0.307**	**0.022**
FDP	−0.171	0.26	−0.049	0.72
**total bilirubin**	**−0.311**	**0.035**	0.05	0.72
UA	−0.147	0.33	0.079	0.57
hsCRP	−0.013	0.93	0.146	0.29

ρ: Spearman’s correlation coefficient, significant *p* values are shown in bold type.

**Table 4:** Results of regression analysis (schizoid personality score as dependent variable).

Independent Variable	Schizoid Personality Score
	Beta
-	-
model	-
**LDL-C**	**−0.312**
model	***N* = 55, AdjR^2^ = 0.08, F(1,53) = 5.714,** ***p* = 0.02, condition index = 8.825**

Significant *p* value is shown in bold type.

**Table 5:** The relationships between serum LDL-C levels and the results of a PC-based trust game.

	Serum LDL-C Levels			
	Male		Female	
	r, ρ	*p*	r, ρ	*p*
Monetary Scores to				
all partners	0.351	0.15	0.379	0.18
male partners	0.378	0.12	0.290	0.32
female partners	0.311	0.21	0.455	0.10
**Trustworthiness of**				
all partners	0.387	0.11	0.474	0.087
male partners	0.351	0.15	0.367	0.20
**female partners**	0.374	0.13	**0.546**	**0.043**
Attractiveness of				
all partners	0.455	0.058	0.246	0.40
male partners	0.445	0.064	0.057	0.85
female partners	0.443	0.065	0.418	0.14

r: Pearson’s correlation coefficient. ρ: Spearman’s correlation coefficient, significant *p* values are shown in bold type.

## Data Availability

The data presented in the present study are available on request from the corresponding author.
